# Factors Related to Low COVID-19 Vaccination Rate in Pregnant and Postpartum Women with and without COVID-19

**DOI:** 10.1055/s-0043-1772589

**Published:** 2023-11-29

**Authors:** Dilek Menekse Beser, Derya Uyan Hendem, Deniz Oluklu, Ezgi Turgut, Necati Hancerliogullari, Sule Goncu Ayhan, Ozlem Moraloglu Tekin, Dilek Sahin

**Affiliations:** 1 Division of Perinatology, Department of Obstetrics and Gynecology, Turkish Ministry of Health Ankara City Hospital, Ankara, Turkey.; 2 Department of Obstetrics and Gynecology, University of Health Sciences, Turkish Ministry of Health Ankara City Hospital, Ankara, Turkey.; 3 Division of Perinatology, Department of Obstetrics and Gynecology, University of Health Sciences, Turkish Ministry of Health Ankara City Hospital, Ankara, Turkey.

**Keywords:** COVID-19 vaccine, postpartum women, pregnancy, vaccine acceptance, vaccine hesitancy

## Abstract

**Objective** This study focused on pregnant and postpartum women during the COVID-19 pandemic, aiming to determine the attitudes and behaviors of vaccinated and unvaccinated groups, and the vaccination behaviors in the groups with and without the disease. The reasons for refusing the vaccine were also questioned.

**Methods** This cross-sectional study was performed from September 2021 to October 2021. The study data were collected using a face-to-face questionnaire. The participants were pregnant women who applied to the hospital for routine antenatal care and were hospitalized, and women in the postpartum period. Additionally, pregnant and postpartum patients who were diagnosed with COVID-19 at the time of admission and were hospitalized and admitted to the intensive care unit due to this disease were also included in the study.

**Results** A total of 1,146 pregnant and postpartum women who completed the questionnaire were included in our study. Only 43 (3.8%) of the participants were vaccinated; 154 (13.4%) of the participants had comorbidities. The number of COVID-19-positive patients was 153. The lack of sufficient information about the safety of the COVID-19 vaccine is the most common reason for the refusal.

**Conclusion** Vaccine refusal can significantly delay or hinder herd immunity, resulting in higher morbidity and mortality. Considering the adverse effects of COVID-19 on pregnancy, it is essential to understand pregnant and postpartum women's perceptions toward vaccination to end the pandemic.

## Introduction

The coronavirus disease 2019 (COVID-19) is considered one of the most widespread, global public health crises due to being one of the leading causes of death internationally. 1 While this disease may have a mild process in pregnant women, severe illness with hospitalization, admission to the intensive care unit (ICU), mechanical ventilation, or death were seen. Pregnant and postpartum women are more vulnerable to developing severe symptoms of infection because of the physiological changes in the immune system that occur during pregnancy. 2 Furthermore, the COVID-19 virus can potentially alter immunological responses at the maternal–fetal interface, affecting both mother and baby. 3 Thus, pregnant women with COVID-19 are at increased risk of adverse pregnancy outcomes. 4 5

The pandemic has weakened healthcare systems, disrupted supply chains, and sparked a mental health crisis; thus, it caused significant public health problems. Although two years have passed since the identification of the disease, an effective and safe treatment has not been found during pregnancy. Vaccination is the best way to protect women and babies against the risks of COVID-19. By the end of 2020, vaccines that became available in many parts of the world were considered the most promising attempt to prevent SARS-CoV-2 infection and defeat the pandemic). 6 Several vaccines have been developed rapidly and authorized for use in many countries. None of the COVID-19 vaccines contained live viruses, thus indicating their suitability for pregnant and postpartum women. Many studies have shown that the vaccines do not raise any concerns about the safety of female reproduction, intrauterine or postnatal development, and their safety and efficacy in pregnancy. 7 8 Therefore, many authorities recommend vaccination. 9 10

Vaccination is considered a keystone, along with other interventions, to overcome the pandemic. However, vaccine hesitancy – defined as the rejection or delayed vaccine acceptance – has the potential to hinder this attempt and is considered to be a significant global health threat. 11 12 It was thought that without a general approach to acceptance by the public, COVID-19 vaccines would not defeat the pandemic. Therefore, vaccination willingness and hesitancy among different populations have been studied since the early process of availability. 13 14 15 According to the results of these studies, it has been observed that pregnant women are more worried about vaccination than the general population. There is a lot of misinformation and concerns about vaccines, especially since pregnancy was excluded from studies in the past years. Moreover, this hesitancy is affected by various factors in vulnerable populations. Therefore, it is important to understand the factors affecting vaccine acceptance and hesitation in pregnant and postpartum women, who are more vulnerable than the general population.

This study aimed to determine the vaccination status of pregnant and postpartum women, the attitudes and behaviors of vaccinated and unvaccinated groups, and the vaccination behaviors in the groups with and without COVID-19. The reasons for refusal to be vaccinated were also questioned.

## Methods

This cross-sectional study was performed in Ankara City Hospital from September 2021 to October 2021. This tertiary hospital is a significant pandemic center with approximately 20,000 births per year. The participants of this study were pregnant women who were were hospitalized during routine antenatal appointments, and women in the postpartum period. Additionally, pregnant and postpartum patients who were diagnosed with COVID-19 at the time of admission and were hospitalized due to COVID-19 and admitted to the ICU were also included in the study. Written informed consent was obtained from all subjects. The applied protocol was approved by the Medical Research Ethics Department of the hospital (E2–21–820).

The study's data were collected using a face-to-face questionnaire by three maternal-fetal specialists. There was no accompanying person with the participants, so no one was affected by their decision at the time of the questionnaire. The patients have similar characteristics regarding religion, language, and race. All the patients had information about how to get the vaccine, whether it was cost-free, and in which centers it could be administered. All pregnant and postpartum women were again informed about the vaccination program of the societies and the Turkish Ministry of Health. All participants had the autonomy to get vaccinated without permission from their husbands or any family member.

Vaccination guidelines were created according to the careful investigation of studies, statements, and assessments performed throughout the world and within Turkey by the Coronavirus Scientific Committee of the Turkish Ministry of Health. It is applied by offering inactive virus and mRNA COVID-19 vaccine options, including for pregnant and postpartum women. If a person wants to be vaccinated, they can book an appointment after checking eligibility online. The COVID-19 vaccines are administered free of charge, mainly at the Family Health Centers and public and private hospitals where the Provincial Health Directorates provide vaccination services. Immunization teams from the Community Health Centers and District Health Directorates provide on-site vaccination services at stations in other public areas. The healthcare staff of the Home Healthcare System administers the vaccines to individuals with their home addresses recorded.

The first section of this study's questionnaire determined maternal characteristics, including age, parity, comorbidities, and sociodemographic characteristics. The second part focused on data about COVID-19 vaccination, the type of vaccine, and gestational age at vaccination. Regarding COVID-19 vaccination, the participants were divided into two groups, vaccinated and unvaccinated. The participants were also asked about their previous vaccination status and their attitudes toward pregnancy tetanus and influenza vaccination. Additionally, questions about COVID-19 contact were asked. The status of relatives regarding the disease and vaccination and whether the participant was encouraged to vaccinate were evaluated. The participants who were not vaccinated were asked about their reasons in the third part of the questionnaire.

Furthermore, COVID-19 infection in patients was confirmed by reverse-transcription polymerase chain reaction (RT-PCR) testing. Patients who were intubated due to severe infection could not be included in the study because they could not complete the questionnaire. Refugees were not included in the study because of language problems.

Statistical analyses were performed using the Statistical Package Social Sciences (SPSS, Inc., Chicago, IL, USA) software version 17. Categorical data were expressed as numbers (percentages) and compared with the Chi-square test in two independent groups. Numerical data were shown in mean ± standard deviation (SD) and median (minimum-maximum) A type-1 error below 0.05 was considered statistically significant. Descriptive statistics, proportions, frequency distribution, and mean values were calculated, and the findings were presented in text, tables, and figures.

## Results

**Fig. 1:**
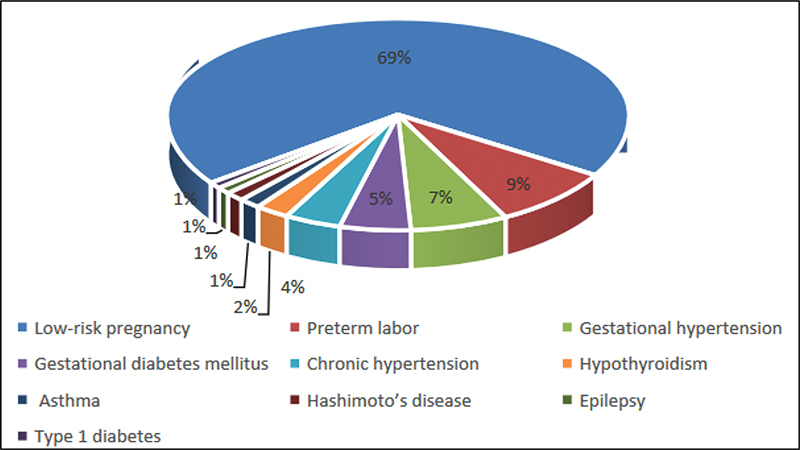
Distribution of high-risk pregnancies among all participants.

In total, 1,146 pregnant and postpartum women who completed the questionnaire were included in this study. The vaccination rate among the participants was 3.8%. Sociodemographic features are shown in Table 1 . The participants were mostly pregnant women in the third trimester. There were 154 (13.4%) participants with comorbidities diagnosed before pregnancy: chronic hypertension ( *n* = 45), hypothyroidism ( *n* = 27), asthma ( *n* = 17), Hashimoto disease ( *n* = 15), epilepsy ( *n* = 11), and type 1 diabetes ( *n* = 10). Patients followed up due to high risk during antenatal care included women with threatened preterm labor ( *n* = 98), gestational hypertension ( *n* = 78), and gestational diabetes mellitus ( *n* = 57). Both comorbidity and risk during antenatal care were considered as high-risk pregnancies. The distribution of these patients among all participants is shown in Fig. 1 .

**Table 1:** Sociodemographic Data

Variables	All participants ( *n* = 1146)
Age	28.1 ± 5.3
Gravidity	2 (1–8)
Parity	1 (0–5)
Gestational week	33 (5–42)
First trimester	44 (3.8%)
Second trimester	217(18.9%)
Third trimester	637 (55.6%)
Postpartum period	248 (21.7%)
Day after birth ( *n* = 248)	3 (0–18)
Number of householders	3 (2–12)
Number of school-age children	1 (0–5)
Number of the person with comorbidity	0 (0–2)
Number of >65-yeard-old householders	0 (0–2)
Income (month, Turkish Lira)	4,216 ± 1,615
Length of hospital stay (days) ( *n* = 117)	6 (1–22)
Comorbidity	154 (13.4%)
High-risk pregnancy	483 (42.2%)
Education status	
None	45 (3.9%)
Primary school	168 (14.7%)
Secondary school	572 (49.9%)
University	361 (31.5%)
Career	
Housewife	873 (76.2%)
Government official	127 (11.1%)
Private sector	26 (2.3%)
Worker	120 (10.5%)
Husband career	
Worker	402 (35.1%)
Government official	270 (23.6%)
Merchant	166 (14.5%)
Private sector	93 (8.1%)
Unemployed	215 (18.8%)

**Notes:**^†^ Values are given as mean ± standard deviation and median (min–max) or as number (percentage).

The responses of all participants and the statistical significance of the answers in vaccinated and unvaccinated women are shown in Table 2 . If the influenza vaccine was recommended during this pregnancy, the rate was significantly higher in the vaccinated group (58.1%, *p* = 0.018). Also in the same group, getting the influenza vaccine during this pregnancy was higher than in the unvaccinated group (7%, *p* = 0.020). The fear of the vaccine harming the baby was higher in the unvaccinated group (69.6%). Hospitalization due to COVID-19 was 2.3% in the vaccinated group and 10.5% in the unvaccinated group. There were 12 patients admitted to the intensive care unit due to COVID-19, and all were unvaccinated. The number of COVID-19-positive patients at the time of inclusion in the study was 153 (13.4%). Among them, those who were vaccinated were only 4. The attitude of these patients toward vaccination was registered in Table 3 .

**Table 2:** Answers of all participants with statistical significance comparison of the answers

Questions	Answers	Participants ( *n* = 1,146)	Vaccinated n = 43 (3.8%)	Not vaccinated n = 1,103 (96.2%)	*p* -value
Did you ever get vaccinated?	Yes	990 (86.4%)	36 (83.7%)	954 (86.5%)	0.603
	No	156 (13.6%)	7 (16.3%)	149 (13.5%)	
Did you get vaccinated in the last five years?	Yes	923 (80.5%)	39 (90.7%)	884 (80.1%)	0.086
	No	223 (19.5%)	4 (9.3%)	219 (19.9%)	
Is the influenza vaccine recommended in this pregnancy?	Yes	46 (4%)	4 (9.3%)	42 (3.8%)	0.090
	No	1,100 (96%)	39 (90.7%)	1060 (96.2%)	
If the influenza vaccine was recommended, would you get vaccinated in this pregnancy?	Yes	466 (40.7%)	25 (58.1%)	441 (40%)	**0.018**
	No	679 (59.3%)	18 (41.9%)	661 (60%)	
Did you get the influenza vaccine during this pregnancy?	Yes	16 (1.4%)	3 (%7)	13 (%1.2)	**0.020**
	No	1,130 (98.6%)	40 (93%)	1,090 (98.8%)	
Is the tetanus vaccine recommended in this pregnancy?	Yes	918 (80.1%)	33 (76.7%)	885 (80.2%)	0.574
	No	228 (19.9%)	10 (23.3%)	218 (19.8%)	
Did you get the tetanus vaccine during this pregnancy?	Yes	879 (76.7%)	33 (76.7%)	849 (77%)	0.273
	No	267 (23.3%)	10 (23.3%)	254 (23%)	
Do you prefer your baby to be vaccinated?	Yes	1,122 (97.9%)	41 (95.3%)	1,081 (98%)	0.228
	No	24 (2.1%)	2 (4.7%)	22 (2%)	
Do you have a high risk of COVID-19 transmission at work?	Yes	92 (8%)	3 (7%)	89 (8.1%)	0.796
	No	1,054 (92%)	40 (93%)	1,014 (91.9%)	
Did you have close contact with COVID-19 positive person?	Yes	330 (28.8%)	11 (25.6%)	319 (28.9%)	0.635
	No	816 (71.2%)	32 (74.4%)	784 (71.1%)	
Has your husband gotten vaccinated for COVID-19?	Yes	812 (70.9%)	36 (83.7%)	776 (70.4%)	0.05
	No	334 (29.1%)	7(16.3%)	327 (29.6)	
Did you support your relatives in getting the COVID-19 vaccine?	Yes	595 (51.9%)	40 (93%)	555 (50.3%)	**<0.001**
	No	551 (48.1%)	3 (7%)	548 (49.7%)	
Have you ever had a relative admitted to ICU due to COVID-19?	Yes	95 (8.3%)	13 (30.2%)	82 (7.4%)	**<0.001**
	No	1,051 (91.7%)	30 (69.8%)	1,021 (92.6%)	
Have you had a relative who died due to COVID-19?	Yes	32 (2.8%)	0	32 (2.9%)	0.629
	No	1,114 (97.2%)	43 (100%)	1,071 (97.1%)	
Positive COVID-19 status now?	Yes	153 (13.4%)	4 (%14,8)	149 (13.5%)	0.426
	No	993 (86.6%)	39 (%85,2)	954 (86.5%)	
Have you been hospitalized due to COVID-19?	Yes	117 (10.2%)	1 (2.3%)	116 (10.5%)	0.082
	No	1,029 (89.8%)	42 (97.7%)	987 (89.5%)	
Have you been admitted to ICU due to COVID-19?	Yes	12 (1%)	0	12 (1.1%)	0.999
	No	1,134 (99%)	43 (100%)	1,091 (98.9%)	
Do you think that the vaccine is harmful to you?	Yes	37 (3.2%)	2 (4.7%)	35 (3.2%)	0.646
	No	1,109 (96.8%)	41 (%95.3)	1,068 (96.8%)	
Do you think the COVID-19 vaccine will harm your baby?	Yes	774 (67.5%)	6 (14%)	768 (69.6%)	**<0.001**
	No	372 (32.5%)	37 (86%)	335 (30.4%)	

**Abbreviation:** COVID-19, coronavirus disease 2019; ICU, intensive care unit. **Notes:** ^†^ Chi-square test. ^‡^ The bold characters were used to define the significant *p* -values < 0.05. ^¶^ Values are given as numbers (percentage).

**Table 3:** Attitudes to vaccines between COVID-19 positive and negative groups

Questions	Answers	Positive n = 153 (13.4%)	Negative n = 993 (86.6%)	*p* -value
Has your husband gotten vaccinated for COVID-19?	Yes	105 (68.6%)	707 (71.2%)	0.515
	No	48 (31.4%)	286 (28.8%)	
Did you support your relatives in getting the COVID-19 vaccine?	Yes	94 (61.4%)	501 (50.5%)	**0.011**
	No	59 (38.6%)	548 (%49.5%)	
Have you ever had a relative admitted to ICU due to COVID-19?	Yes	29 (19%)	66 (6.6%)	**<0.001**
	No	124 (81%)	927 (93.4%)	
Have you had a relative who died due to COVID-19?	Yes	12 (7.8%)	20 (2%)	**<0.001**
	No	141 (92.2%)	973 (98%)	
Have you been hospitalized due to COVID-19?	Yes	51 (33.3%)	66 (5.6%)	**<0.001**
	No	102 (66.7%)	927 (93.4%)	
Have you been admitted to ICU due to COVID-19?	Yes	9 (5.9%)	3 (0.3%)	**<0.001**
	No	144 (94.1%)	990 (99.7%)	

**Abbreviations:** COVID-19, coronavirus disease 2019; ICU, intensive care unit. **Notes:** ^†^ Chi-square test. ^‡^ The bold characters were used to define the significant *p* -values < 0.05. ^¶^ Values are given as a number (percentage).

Although there is no statistical significance, the vaccination rate of the participants' partners was also higher in the COVID-19 negative group than in the positive one (71.2% vs. 68.6%). The overall vaccination rate in the study was 3.8%. Participants were vaccinated mainly in the second trimester (37.2%). The pregnancy periods during which they received the vaccine was 16.3% before pregnancy, 9.3% in the first trimester, 37.2% in the second trimester, and 25.6% in the third trimester. The most common vaccine was the single-dose BNT162b2 mRNA (Pfizer-BioNTech, 19.9%). The reasons for vaccine refusal are shown in Table 4 . The lack of sufficient information about the safety of the COVID-19 vaccine is the most common reason for refusal. Other frequent reasons were worry that it would be harmful to the baby, belief that the vaccine would not work, and family members' refusal to vaccinate.

**Table 4:** Reasons for the COVID-19 vaccine refusal?

Questions	Not vaccinated n = 1,103
Lack of sufficient information about the safety of the COVID-19 vaccine	535 (48.5%)
The vaccine is harmful to my baby	329 (29.8%)
I don't think that vaccine will work	209 (18.9%)
I think the virus will mutate	119 (10.8%)
Family members disagree with the COVID-19 vaccine	114 (10.3%)
The vaccine is harmful to my body	94 (%8.5)
The vaccine will cause COVID-19 infection	93 (8.4%)
COVID-19 is not a severe disease	68 (6.2%)
I have a low risk of getting a COVID-19 infection	67 (6.1%)
I believe that even if I am sick, my baby and I will not encounter any adverse events	55 (5%)
Afraid of injection	33 (3%)

**Abbreviations:** COVID-19, coronavirus disease 2019. **Notes:** † Values are given as a number (percentage).

## Discussion

The present study showed a very low vaccination rate of 3.8% in pregnant and postpartum women. This low vaccination rate was unexpected for us. In a previous study from our clinic, before the vaccination began, 37% of pregnant women had the intention to get the vaccine if it was offered during pregnancy. 16 In another study that showed vaccine acceptance in postpartum women, 33.3% of participants were accepting. 17 Despite the acceptance rates in our previous studies, vaccination during pregnancy and postpartum was relatively low in the present study. In our previous study with pregnant women, the group that accepted the vaccine thought they were sufficiently informed about the COVID-19 vaccine compared with the group that refused it. In the group with vaccine refusal, the most common reasons for rejection were lack of knowledge and worry that it might be harmful to the baby. The most common reasons for vaccine refusal in postpartum women were insufficient knowledge about the application to women in this period and doubts over its effectiveness. Similar to these studies, we found that the lack of knowledge about vaccines and the belief that they would harm the baby were the majority of the reasons for low vaccination acceptance in pregnant and postpartum women. These results show the need to fight not only the pandemic but also incomplete or wrong information, and knowledge must be disseminated.

Pregnant women are notably vulnerable to infectious diseases due to changes in immunity and respiratory and cardiovascular physiology that happen during pregnancy. The Centers for Disease Control (CDC) data and other publications showed that pregnant women were three times more likely to be admitted to the ICU or need intubation and 1.5 times more likely to die from COVID-19 than nonpregnant women. 18 A comprehensive study from our clinic, a tertiary pandemic center sharing experiences of pregnant women with COVID-19 and comparing clinical outcomes of pregnancy trimesters, showed that pregnant women are at higher risk of developing severe illnesses and complications. 5

In the present study, 13.4% of the participants had comorbidities. Although it is well known that individuals with chronic illness have a higher risk of severe disease and death, those participants still avoided getting vaccinated. We believe this is a very dramatic finding.

The COVID-19 vaccination process in Turkey started in January 2021. Vaccinations are given free of charge to determined age groups through appointments at family health centers and hospitals. According to this procedure, COVID-19 vaccination can be applied before, during, and after pregnancy. A previous study showed that the rates of COVID-19 vaccine acceptance among pregnant women vary significantly, such as 28.8 to 84.4% according to Turkey's official data. 19 Despite the guidelines and recommendations, pregnant and postpartum women's vaccination rate was relatively low in the present study.

Vaccination is the only way to defeat the pandemic. Although medical organizations and committees recommend vaccination for pregnant and lactating women, the rates are still low. In clinical studies, the vaccine has shown a >85% reduction in symptomatic COVID-19 and risk of transmission. 20 21 In our research, the number of patients with the disease was 153 (13.4%).

In studies conducted before the COVID-19 outbreak, gender-related vaccination challenges affected populations. 22 23 Women were less likely than men to receive relevant or reliable information due to a lack of education and access to information, as well as work and home care obligations. Additionally, women were less reliant on vaccines and were less able to make health-related decisions due to limited household decision-making power. Furthermore, they had more difficulty reaching vaccination locations due to limited mobility. All of the patients in our study group were informed about how to access the vaccine, and that it is cost-free and can be immediately included in a vaccination program by their health provider if they so desired. Understanding how genders norms and power dynamics affect admission and requests for vaccination in different conditions is crucial for extending vaccine access. Gender-related barriers should be considered when planning and expanding vaccine distribution to reach all populations.

Vaccine hesitancy is a global problem, posing a significant threat to controlling the COVID-19 pandemic. 24 Previous studies have determined several factors associated with this disease's vaccine hesitancy. 25 26 Socioeconomic and demographic characteristics (age, gender, income, occupation, and marital status), incomplete or incorrect information about vaccines, religious beliefs, confidence in the content of vaccines, and possible side effects are some of these factors. Despite vaccine hesitancy, requests have increased over time, and the inequality of access to vaccines within and between countries is remarkable. It is due to vaccination that diseases such as smallpox, poliomyelitis, and yellow fever, which used to cause millions of deaths and disabilities in many parts of the world, are now almost completely extinct. In light of this information, we believe that if COVID-19 vaccines are optimally and equitably received worldwide, they could have a similar impact on the pandemic.

The COVID-19 pandemic has led to the administration of “social distancing” strategies, which are critical to limiting the spread of the virus, but this situation had some psychological consequences. A recent study examined the effects of loneliness and social distance on human health. 27 Furthermore, a previous study found increased anxiety levels in high-risk pregnant women compared with normal pregnancies during the COVID-19 pandemic. 28 Mortazavi et al. suggest that health professionals can reduce anxiety levels by supporting pregnant women and improving their well-being. 29 In addition to this support, COVID-19 vaccination can also reduce anxiety. Family members' decisions about vaccination influence the patients' decisions; therefore, it is essential to support pregnant women's decisions in this situation. 19 According to our study, in the vaccinated group, the COVID-19 vaccination rate of their husbands was higher than in the unvaccinated one (83.7 vs. 70.4%). We found that the rate of supporting relatives for vaccination was significantly higher in the COVID-19-positive group. In this group, the rate of mortality and ICU admittance among relatives because of COVID-19 was also higher. Accordingly, these results may indicate the influence of experiencing unfavorable situations in understanding the severity of the disease.

Counseling on vaccination should include the disease's risks and the benefits of vaccination before, during, or after pregnancy, while breastfeeding. Insufficient data on vaccine safety was the main reason participants were against it. This result was similar to a previous study on COVID-19 vaccine acceptance before the beginning of the vaccination program. 16

A lack of sufficient research on the effects of these vaccines in pregnancy could influence the vaccination rate in pregnant and postpartum women. No side effects of the vaccine have been reported among women who participated in clinical trials in the early stages of vaccine testing and became pregnant at the end. 30 31 Nevertheless, the worry that it could harm the baby was higher in the unvaccinated group.

It is essential to build public confidence in vaccination with consistent communication programs by the government. At the same time, detailed information about the dangers of the disease should be given through effective vaccination campaigns. It has been shown that COVID-19 causes many adverse pregnancy outcomes, such as preterm birth and miscarriage. 5 While the maternal and fetal effects of COVID-19 have proven so much, the reasons for vaccine refusal in pregnant and postpartum women should be investigated further. Future studies should focus on the reason for this low vaccination rate, whether reporting the adverse outcomes of COVID-19 is enough, or whether further explanations are needed about the positive effects of the COVID-19 vaccine. A previous study about the tetanus vaccine showed a high vaccination rate in pregnant women who were well informed about vaccination. 32 To ensure herd immunity, the effects of vaccines and the necessary doses for protection should be explained to the public in detail. Reliable health communication and encouraging the public about vaccination can influence positive health behaviors. Lessons learned from previous epidemics of infectious diseases, such as HIV, H1N1, and SARS, have shown us the importance of reliable sources of information in the fight against infections.

The main strengths of the present study were the large number of participants, which included patients who were COVID-19 positive, as well as the vaccination rates in high-risk pregnancies. The study's main limitation was the short study period.

## Conclusion

Vaccine refusal can significantly delay or hinder herd immunity, resulting in more significant rates of morbidity and mortality. Considering the adverse effects of COVID-19 on pregnancy, it is essential to understand pregnant and postpartum women's perceptions toward vaccination to end the pandemic.
